# Controlled release of nitric oxide for enhanced tumor drug delivery and reduction of thrombosis risk†

**DOI:** 10.1039/d2ra05438h

**Published:** 2022-11-11

**Authors:** Rui Liu, Baofeng Xu, Zhifang Ma, Hongbo Ye, Xinghua Guan, Yue Ke, Zehong Xiang, Qiang Shi

**Affiliations:** Department of VIP Unit, China-Japan Union Hospital of Jilin University Changchun Jilin 130022 China; Stroke Center and Department of Neurology, First Hospital of Jilin University Changchun Jilin 130022 China; State Key Laboratory of Polymer Physics and Chemistry, Changchun Institute of Applied Chemistry, Chinese Academy of Sciences Changchun Jilin 130022 China shiqiang@ciac.ac.cn zfma@ciac.ac.cn; University of Science and Technology of China Hefei Anhui 230026 China

## Abstract

Platelets activation and hypercoagulation induced by tumor cell-specific thrombotic secretions such as tissue factor (TF) and cancer procoagulant (CP), microparticles (MPs), and cytokines not only increase cancer-associated thrombosis but also accelerate cancer progress. In addition, the tumor heterogeneity such avascular areas, vascular occlusion and interstitial fluid pressure still challenges efficient drug delivery into tumor tissue. To overcome these adversities, we herein present an antiplatelet strategy based on a proteinic nanoparticles co-assembly of l-arginine (LA) and photosensitizer IR783 for local NO release to inhibit the activation of tumor-associated platelets and normalize angiogenesis, suppressing thrombosis and increasing tumoral accumulation of the nanoagent. In addition, NIR-controlled release localizes the NO spatiotemporally to tumor-associated platelets and prevents undesirable systemic bleeding substantially. Moreover, NO can transform to more cytotoxic peroxynitrite to destroy cancer cells. Our study describes an antiplatelet-directed cancer treatment, which represents a promising area of targeted cancer therapy.

## Abstract 2

Antiplatelet nanoparticles co-loaded with l-arginine (LA) and photosensitizer IR783 were established for local NO release to inhibit tumor-associated platelets activation, suppress thrombosis and increase tumoral accumulation of the nanoagent.

## Graphical abstract

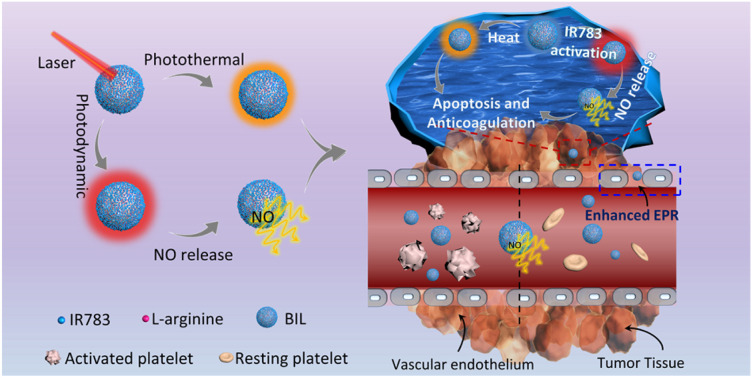

## 1. Introduction

Thrombosis is the most frequent complication and the second commonest cause of death in patients with malignant disease due to the pathogenesis of blood coagulation activation.^1–4^ Tumor cell-specific thrombotic secretions such as tissue factor (TF) and cancer procoagulant (CP), microparticles (MPs), and cytokines significantly contribute to the hypercoagulable state and high risk of thrombosis.^5–7^ Reciprocally, this prothrombotic state parallels the development of malignancy and supports the malignant process by promoting cancer growth, maintenance and propagation. In particular, hyperreactivity of platelets is one of the direct mechanisms of cancer-associated thrombosis and is involved in the progression of cancer.^8–10^ The cross-talk between cancer cells and activated platelets directly impacts tumor growth, tethering and spread through myriad growth factors from their dense and α-granules and peroxisomes. What's more, platelets can be recruited to shroud tumor cells, thereby shielding them from immune responses, and facilitating cancer growth and dissemination. Therefore, blocking the activation cascade of coagulation and platelets may offer a means to reduce both the risks of development and progression of cancer and the risk of thrombosis. The research gap on breaking this vicious activation loop has been barely addressed so far.

Despite being highly permeable to molecular drugs and nanomedicines because of large gaps between vascular endothelial cells, the so called enhanced permeability and retention (EPR) effect, efficient drug delivery is still challenged by tumor heterogeneity such avascular areas, vascular occlusion or embolization, and interstitial fluid pressure.^11–13^ Therefore, impaired tumor blood flow that overcomes tumor heterogeneity is indeed critical. Nitric oxide (NO), a highly potent endogenous messenger molecule, has been extensively studied for its crucial role in physiological and pathological processes, such as cancer, inflammatory disorders, platelets regulation and thrombus formation.^14–17^ In particular, NO is reported to regulate tumor blood flow by normalized angiogenesis, improvement of the interstitial fluid pressure and prevention of vascular occlusion, thus elevating therapeutic agent accumulation within the solid tumor.^18–22^ Moreover, NO can cause DNA damage, apoptosis, and cell death *via* the formation of toxic peroxynitrite induced by reaction between over-expressed ROS and NO in tumor cells.^23,24^ In addition, nitric oxide prevents platelet adhesion to the vessel wall and provides a negative feedback mechanism for thrombus formation.^25^ NO may inhibit the active conformation of glycoprotein IIb/IIIa and expression of P-selectin and decrease recruiting of more platelets and association with fibrinogen, blocking the thrombus formation. More importantly, it is reported that the endogenous NO can down-regulate the expression of tissue factor (TF), the major initiator of the hypercoagulation state in cancer patients.^26^ Based on these above advantages, establishment of NO-generating agents may augment the EPR effect and the anticancer effects of nanomedicines and meanwhile prevent platelets activation, hypercoagulation and thrombus formation.

**Scheme 1:**
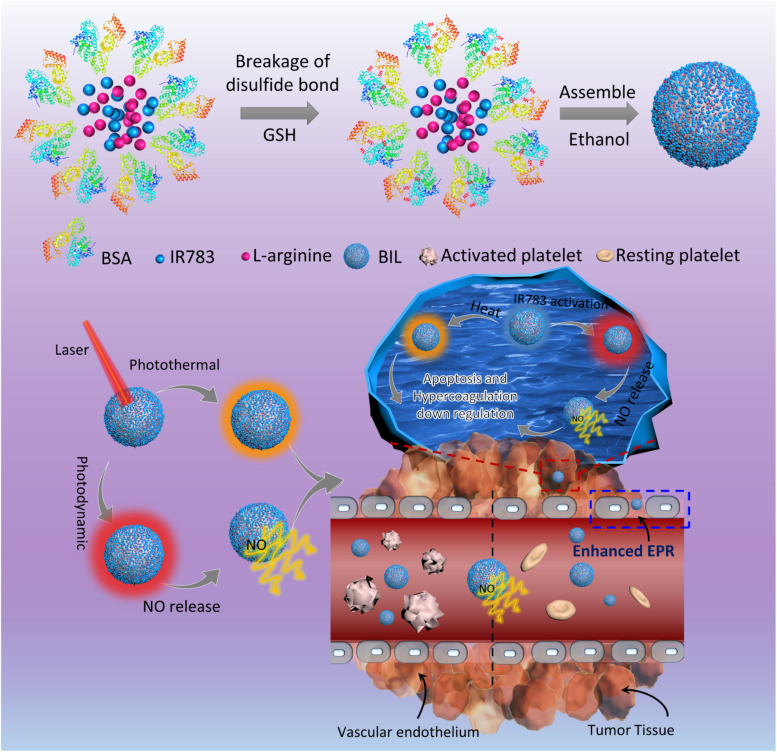
Schematic diagram for the antiplatelet nanosystem. IR783 was first activated by NIR light and transfers energy or electrons to oxygen molecules and other substrates to produce ROS, which next oxidize LA to form citrulline and NO. After intravenous administration, NO first blocks the activation of platelets, augmenting perfusion of the particle into tumor tissues. On the other hand, the NO released from the internalized particles within cancer cells not only promotes the apoptosis of cells but also downregulates the hypercoagulation induced by cancer cells, which in turn prevents the activation of platelets.

In the present work, we designed a NO-triggering system based on LA as the NO precursor, inspired by the *in vivo* NOS/NO pathway, to achieve the controlled release of NO for the local inhibition of tumor-associated platelets activation (Scheme 1). The released NO from LA oxidation activates the soluble guanylyl cyclase (GC) and then downregulates the activity of platelets and the thrombosis risk for cancer patients.^27–30^ In brief, LA and IR783 were first co-loaded on the scaffold of bovine serum albumin (BIL). In this system, LA molecules serve as the nitric oxide precursor mediated by the photodynamic process of photosensitizer IR783 under stimulation of a near infrared (NIR) laser. The laser-activated IR783 transfers its energy or electrons to oxygen to generate ROS, which then induce guanidino oxidation of LA to release NO, increasing the local NO concentration within the tumor tissue. Firstly, the induced NO can inhibit the platelets activation to suppress the hypercoagulation and thrombosis risk originally induced by tumor cells. In addition, the NO can be transformed to more toxic peroxynitrite which helps to destroy the tumor tissue. We prepared the NIR laser responsive nanosystem and investigated the NO release efficacy, antiplatelet capability and enhanced nanoparticle retention in tumor tissues. This work provided a new insight into cancer therapy based on the tumor-local release of NO controllably and is significantly promising for the development of an antiplatelet-assisted therapy strategy.

## 2. Experimental section

### 2.1. Materials and reagents

Bovine serum albumin (BSA), ethanol, l-arginine hydrochloride and IR783 were obtained from Aladdin Reagent (Shanghai) Co., Ltd. Penicillin–streptomycin (100×) was obtained from Life Technologies Corporation (LA, USA). Standard fetal bovine serum (FBS) was purchased from Tianjin Haoyang Biological Manufacture Co., Ltd (Tianjin, China). Fluorescein diacetate (FDA) and propidium iodide (PI) were purchased from Sigma-Aldrich (MO, USA). l-Arginine ELISA kits were purchased from Shanghai Enzyme-linked Biotechnology Co., Ltd. Griess reagent kit and DAF-FM DA were obtained from Beyotime Biotechnology.

### 2.2. Preparation of BIL nanocomplex

The BIL was synthesized by co-assembly of BSA, IR783 and LA according the literature.^31–33^ Typically, BSA (80 mg), GSH (75 mg), IR783 (20 mg) and LA (20 mg) were dissolved in 5 mL of PBS at pH = 7.4 under stirring. Then the mixture was sonicated for 5 min and stirred for another 30 min. Subsequently, ethanol (2 mL) was dropped into the above solution under stirring. After 0.5 h, the reaction mixture was dialyzed (MWCO 100 000) against deionized water to remove the extra reactants. The BIL composites were collected by lyophilization of the supernatant after centrifugation at 20 000*g* for 10 min (yield: 108 mg). The BSA–IR783 (BI) and BSA–LA (BL) composites were synthesized by the same method without addition of LA or IR783. The morphology was observed by TEM (JEOL JEM-1400, Japan). UV-vis absorption spectra were measured on a LAMBDA 25 spectrometer (PerkinElmer) and the loading amount of IR783 in BIL was determined by UV-Vis absorption spectra (18%, 180 μg mg^−1^). The loading amount of LA was analyzed using a human l-arginine ELISA kit (12%, 120 μg mg^−1^). Zeta potentials and hydrodynamic diameters were measured using a Malvern Zetasizer Nano ZS90 (Malvern Instruments Ltd., UK).

### 2.3. Photothermal effect measurement

To study the photothermal effect of BIL, 1 mL of aqueous dispersions of BIL with different concentrations (IR783 concentration: 0, 80, 120, 160 and 200 μg mL^−1^) in quartz cuvettes were irradiated by a continuous NIR laser (808 nm, 1 W cm^−2^) for 10 min. The temperature of the solution was measured by a digital thermometer with a thermocouple probe and recorded every 10 s. To study the photothermal stability of BIL (667 μg mL^−1^, equal to 120 μg mL^−1^ of IR783) and free IR783 (120 μg mL^−1^ of IR783) aqueous dispersions were continuously exposed to the 808 nm laser at 1 W cm^−2^ for 5 min. Then the solution was cooled to the initial temperature. Then, the irradiation cycle was repeated for five times. The temperature of the solution was recorded every 10 s.

### 2.4. NIR laser mediated NO release assay of BIL

First, we detected the oxidative effect on LA of H_2_O_2_, ˙OH and ˙O_2_^−^, three major types of ROS. The Fenton reaction (Fe^2+^ reacting with H_2_O_2_) and KO_2_ (KO_2_/18-crown-6 (2 : 1) dissolved in DMSO) were chosen to produce ˙OH and ˙O_2_^−^, respectively. Then 4-amino-5-methylamino-2,7-difluorofluorescein diacetate (DAF-FM DA) and 2-phenyl-4,4,5,5-tetramethylimidazoline-3-oxide-1-oxyl (PTIO) were chosen as the NO probe and scavenger. The DAF-FM DA was first mixed with LA, after which the H_2_O_2_, ˙OH and ˙O_2_^−^ solutions were added and the fluorescence in each well was measure by a microplate reader (*λ*_ex_/*λ*_em_ = 490/520 nm). PBS solution served as the control.

Then the NO generation in the photodynamic process was measured. Under 808 nm laser irradiation, the photosensitizer IR783 was first activated to induce the ROS production, which will oxidize the LA to release NO. To measure the NO generation, typically, the BIL (100 μL, 1 mg mL^−1^), BI (100 μL, 1 mg mL^−1^), IR783/LA mixed solution (100 μL, LA: 120 μg mL^−1^, IR783: 180 μg mL^−1^), free LA (100 μL, 120 μg mL^−1^) and free IR783 (100 μL, 180 μg mL^−1^) were added to a 96-well plate, after which Griess Reagent was added to each sample. Then the solution in each well was exposed to the 808 nm laser at a power density of 1 W cm^−2^ for 5 min and the absorbance (*A*_1_) was measured by a microplate reader. The absorbance changes (Δ*A*) for the above solutions without addition of the Griess reagent were measured after exposure to the 808 nm laser (1 W cm^−2^, 5 min) and were subtracted from *A*_1_ for calibration. Then the concentration of NO was read from the premeasured standard curve. All measurements were performed in triplicate. The NO generation induced by BIL under different laser power density (0.25, 0.5 and 1 W cm^−2^) was also measured by the same method as above.

### 2.5. Inhibition of platelets activation by BIL

Platelets were first separated from freshly heparinized blood from the orbit of the same mice strain (20–25 g). Then the platelets were extracted from 1.2 mL of blood spread onto the Petri dishes (1 mL in each dish) after which BIL was added into each dish (1 mg mL^−1^). After 20 min, 20 μM of collagen was dropped into each dish and the mixture in the dish was irradiated with the 808 nm laser for 5 min. Then the aggregation of platelets was observed by microscope.

### 2.6. Study of intracellular generation of NO from BIL

The intracellular generation of NO was investigated using DAF-FM DA. In brief, HeLa cells were seeded into a 96-well plate and divided into three groups: (a) untreated Control group, (b) BIL treated group, and (c) BIL with laser treated group. After 24 h, the medium was replaced by DAF-FM DA (100 μL, 5 μM) and the cells in all groups were exposed to the 808 nm laser at a power density of 1 W cm^−2^ for 5 min. The fluorescence was observed by CLSM (ex: 488 nm; em: 500–580 nm).

### 2.7. Thrombosis targeting in mice tail thrombosis model

All animal procedures were performed in accordance with the Guidelines for Care and Use of Laboratory Animals of Changchun Institute of Applied Chemistry, Chinese Academy of Sciences and approved by the Animal Ethics Committee of Changchun Institute of Applied Chemistry, Chinese Academy of Sciences. The inhibition of tail thrombosis assay in mice was performed by the reported method using carrageenan to induce mouse tail thrombosis.^34,35^ For the thrombus targeting analysis, the free IR783 and BIL were intravenously injected at a concentration of 6 mg kg^−1^, then 1% of carrageenan (50 mg kg^−1^) was administered through an intraperitoneal injection into the mice to induce tail thrombosis. The fluorescence images of mice tails were captured after 24 h.

### 2.8. Antithrombotic assay and tail bleeding assay for tumor-bearing mice

Saline, BI or BIL was injected *via* tail vein into tumor-bearing mice (18–20 g; *n* = 8 per group). After 24 h, the tumor tissues were irradiated by the 808 nm laser for 5 min and the treatment was repeated for 7 cycles. Then the tail thrombosis model was established by intraperitoneal injection of 1% of carrageenan (50 mg kg^−1^). The length of the black appearance in each tail was recorded 24 h post injection.

For the tail-bleeding time measurement, saline, BI or BIL was injected *via* tail vein into tumor-bearing mice (18–20 g; *n* = 8 per group). After 24 h, the tumor tissues were irradiated by the 808 nm laser for 5 min and the treatment was repeated for 7 cycles. Then 2 cm long sections of the tail were amputated and the amputated tail was immediately immersed in 14 mL of pre-warmed sterile saline at 37 °C. The bleeding time was defined as the time required for a wound to stop bleeding for at least 10 s and was recorded with a stopwatch.

### 2.9. Biocompatibility and cancer cell killing effect of BIL *in vitro*

HeLa cells (5 × 10^3^ cells per well) seeded into 96-well plates were incubated in 5% CO_2_ at 37 °C. After 24 h, 100 μL of different concentrations of BIL (0, 25, 50, 100, 200, 400, and 800 μg mL^−1^) were added into each well of the 96-well plate and the cells were continued to be cultured for 24 h. Then 10 μL of CCK-8 was added into each well and the cells were continued to be incubated for 4 h in 5% CO_2_ at 37 °C. The absorbance at 450 nm was recorded using a plate reader and the percentage of cell viability was determined by comparing cells with the untreated control.

HeLa cells (1 × 10^5^ cells per well) were seeded into 96-well plates and incubated in 5% CO_2_ at 37 °C for 24 h. Then the cells were separately treated with free LA, free IR783, BI or BIL (200 μg mL^−1^, 100 μL) for 24 h. Then the cells in all groups were exposed to the 808 nm laser (1 W cm^−2^) for 5 min. After incubation for another 24 h, 10 μL of CCK-8 was added into each well and the cells were continued to be incubated for 4 h in 5% CO_2_ at 37 °C. The absorbance at 450 nm was recorded using a plate reader and the percentage of cell viability was determined by comparing cells with the untreated group. In addition, cell viability was also evaluated using an FDA/PI double staining assay for 15 min and observed under a CLSM.

### 2.10. NO production mediated enhanced accumulation of BIL within tumor tissues

NIR fluorescence imaging was conducted to trace the distribution of BIL and control formulations. In brief, BIL and BI were intravenously injected into mice when the tumor volume reached about 200 mm^3^. Fluorescence images of mice were acquired using a CRI maestro system at 4 h and 24 h post injection.

### 2.11. Animal tumor model and *in vivo* NO-assisted tumor treatment

All animal experiments were carried out using protocols approved by the Ethical Committee of the Changchun Institute of Applied Chemistry, Chinese Academy of Sciences. The *in vivo* tumor therapy model was performed using uterine cervical cancer cells (U14) in female Kunming mice aged 6–8 weeks and weighing 20–25 g. U14 tumor-bearing mice were prepared by subcutaneous injection of around 1 × 10^6^ of mouse U14 cells and the mice were randomly divided into 4 groups: control, free IR783/LA, BI and BIL. When the mice tumor volumes approached around 100 mm^3^, 1 mL of saline, free IR783/LA, BI or BIL was injected *via* the tail vein of each mouse in the above groups every two days and the concentrations of IR783 and LA in the above formulations were equal to 180 μg mL^−1^ and 120 μg mL^−1^. At 24 h post-injection, tumors, except for the control group, were exposed to the 808 nm laser at 1 W cm^−2^ for 5 min. The tumor sizes were measured every 2 days for 2 weeks and the volume was calculated according to the equation: volume = (tumor length) × (tumor width)^2^/2. On the 15th day, all mice were dissected and their tumors and major organs including heart, liver, spleen, lung and kidney were harvested, fixed with 4% paraformaldehyde, sectioned into slices and stained with hematoxylin and eosin (H&E) for histological analysis.

## 3. Results and discussion

### 3.1. Preparation and characterization of BIL

**Fig. 1:**
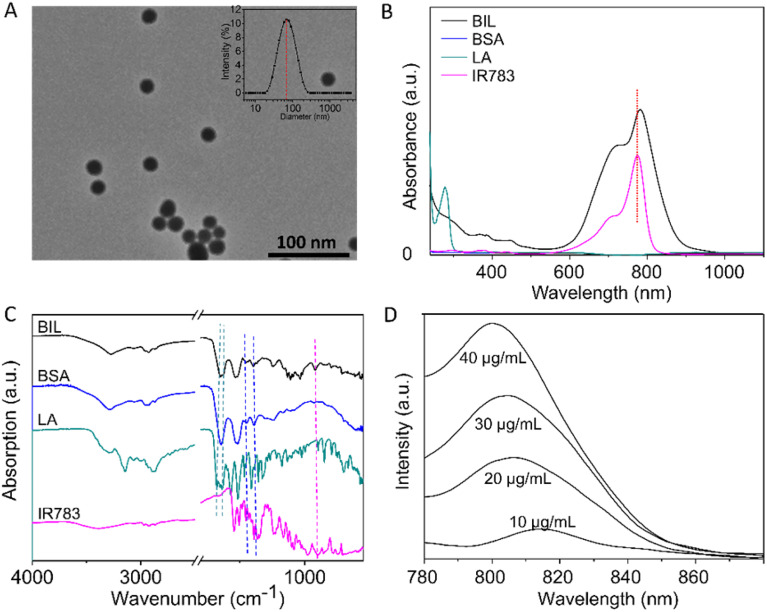
Characterization of BIL. (A) TEM image of BIL (the inset data in the upper-right of the image is the hydrated particle size at 24 h measured using the DLS method). (B and C) Comparison of UV-vis and FTIR spectra of IR783, LA, BSA and BIL. The peak assignments in the FTIR spectra are marked by coloured dotted lines. (D) Fluorescence spectra of different concentrations of IR783 encapsulated within BIL.

Briefly, BIL was prepared by nanoprecipitation of BSA and l-arginine with IR783 encapsulated into the NPs. GSH was applied to break the disulfide bonds and then ethanol was added to promote the assembly of BSA, l-arginine and IR783. As shown in Fig. 1A, the transmission electron microscope (TEM) results of BIL showed a spherical morphology and a relatively uniform size ≈32 nm which is similar to BI and BL (Fig. S1†). The stability of BIL in PBS was measured by the dynamic light scattering (DLS) method over 7 days. The dynamic hydrated diameter was 48 nm determined immediately after synthesis. Then it increased to 66 nm at 24 h caused probably by slightly aggregation and it kept this value during the following 7 days (Fig. S2†). The characteristic peak in UV-vis spectroscopy at 783 nm indicates the successful encapsulation of IR783 (Fig. 1B). As a complement to UV-vis spectroscopy, Fourier transform infrared (FT-IR) spectra were also recorded to verify the co-assembly of BSA, LA and IR783 (Fig. 1C and S3†). The two characteristic peaks of BIL in the spectrum at 1673 and 1633 cm^−1^ can be assigned to the C=N stretching vibration of LA. The acrylamide band I and II of the BSA scaffold were overlapped with the peaks of LA and difficult to tell apart, yet the bands at *ca.* 1386 and 1450 cm^−1^ can be assigned to C=O symmetric stretching of the COO^−^ group and CH_2_ scissoring vibration of BSA, respectively. The peak appearance at 897 cm^−1^ was associated with the Ar–H ring deformation vibration of loaded IR783.^36–41^ Besides, the fluorescence spectra of BIL were recorded, in which the fluorescence intensity at 810 nm (*λ*_ex_ = 760 nm) is proportional to the concentration of IR783 in this concentration range.

### 3.2. Photothermal conversion and NO production of BIL

**Fig. 2:**
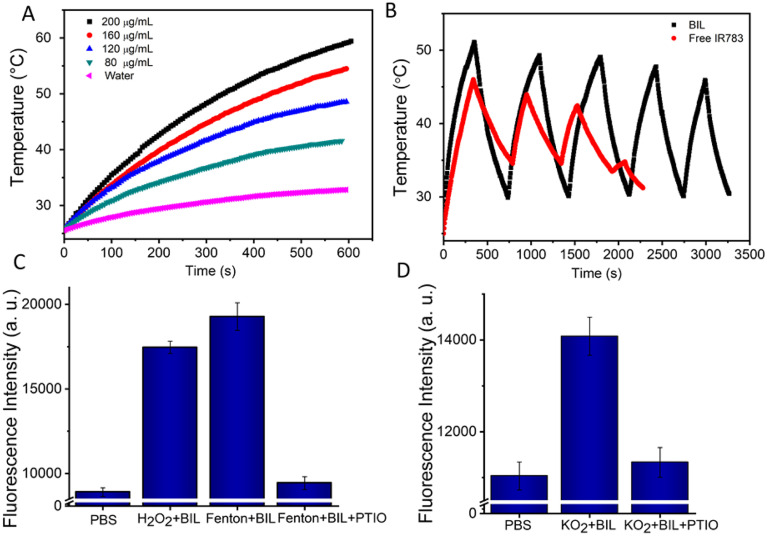
Concentration-dependent photothermal conversion (A) and photostability (B) of BIL. An aqueous dispersion of BIL (1 mL) in a cuvette was irradiated with NIR light (808 nm, 1.0 W cm^−2^) and the temperature was determined using a thermocouple. (C and D) NO generation from LA oxidation by H_2_O_2_, OH˙ and O_2_^−^.

The encapsulated IR783 provides the BIL nanocomplex an excellent photothermal conversion performance. Therefore, we monitored the real-time temperature changes under NIR laser irradiation (808 nm, 1.0 W cm^−2^) for 10 min of the aqueous dispersions of BIL at various concentrations. Remarkably, the temperature of the BIL aqueous solution rapidly increased after irradiation, whereas the temperature of pure water was only elevated by 5.8 °C (Fig. 2A). Moreover, the photothermal effects of BIL exhibited obvious concentration-dependent tendencies with the maximum temperature 58 °C at 200 μg mL^−1^. Next, we investigated the photostability of BIL (Fig. 2B). Compared to free IR783, BIL (IR783: 160 μg mL^−1^) still presented more effective photothermal effects even after five on–off cycles of laser irradiation, with a slight drop in the maximum temperature from 52.3 °C to 46.2 °C. Whereas a rapid attenuation was observed for free IR783, showing the poor photostability. The above results reveal that the synthesized BIL complex has a promising photothermal effects and good photostability, conducive to the following *in vitro* and *in vivo* phototherapy.

Next, the ROS induced NO generation was assessed using the NO probe DAF-FM DA, which binds with NO and emits twenty times amplified fluorescence signal. As reported, there are several types of ROS during the photodynamic process that probably can oxidise LA.^42,43^ The oxidizability of H_2_O_2_, OH˙ and O_2_^−^ towards LA was further measured and the results indicate that the LA can be oxidized by all these types of ROS and transformed to NO at the micromole level (Fig. 2C, D and S4†). The NO produced by BIL increased gradually to 4.1, 7.4 and 14.9 μM within 10 min under different laser powers, indicating an efficient NO release. The morphology characterization revealed no significant degradation for BIL after these photoreactions (Fig. S5†). Remarkably, in the thrombus microenvironment, ROS is a key mediator of platelet activation and aggregation. LA molecules can react with ROS to reduce the ROS concentration and prevent platelet activation. Besides, NO can directly downregulate the platelet activity through the cGMP pathway and helps to inhibit the thrombogenesis.

### 3.3. NO mediated platelet inhibition

**Fig. 3:**
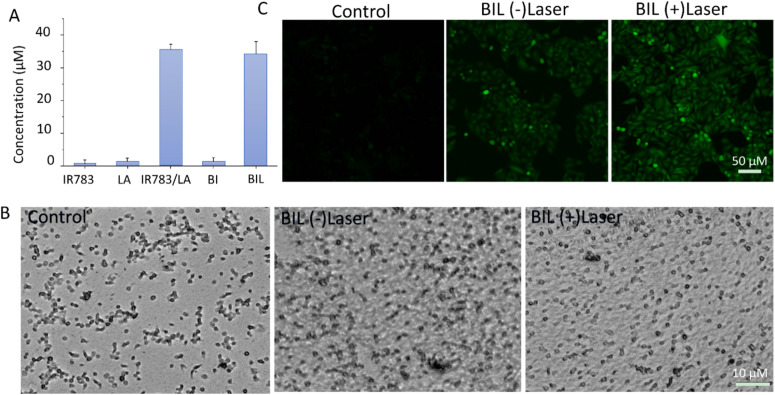
(A) Evaluation of NO production from IR783, LA, IR783/LA, BI and BIL. (B) Inhibition of platelet aggregation by BIL with or without laser treatment. (C) *In vitro* NO production from BIL with or without laser treatment.

NO generation induced by ROS from laser activated IR783 was next detected using the standard Griess assay. As shown in Fig. 3A, there was negligible NO generation from the IR783, LA and BI groups under stimulation of the 808 nm laser. However, IR783/LA and BIL released a large amount of NO after irradiation by the laser compared to the other groups. The results reveal that the synergy between IR783 and LA induces the NO generation, probably due to the oxidation of LA by ROS from laser activated IR783. Next, we further evaluated the inhibition of platelets aggregation by BIL with or without laser treatment (Fig. 3B). The platelets were pre-incubated with BIL and then activated by collagen and irradiated by the laser. The control group shows obvious platelet aggregation after activation. After BIL (without laser) treatment, the platelet aggregation was a little decreased probably due to a small amount of oxidation of LA by H_2_O_2_ from activated platelets. More importantly, BIL with laser treatment significantly inhibited the aggregation. The result reveals that laser stimulation triggers NO production due to the oxidation by ROS from the photodynamic process and can effectively prevent the platelet activation.

It is reported that NO can destroy cancer cells after oxidation by ROS overexpressed by tumor cells and transformation to the more cytotoxic peroxynitrite. Therefore, we detected the intracellular NO generation induced by ROS from PDT (Fig. 3C). The signal in BIL treated cells showed a weak green fluorescence, but it is enhanced after laser irradiation. It indicates that NIR light triggers the NO generation because the laser excites IR783 molecules and leads to the ROS production, which oxidizes the LA molecules to release NO. The weak fluorescence in the BIL (−)laser group probably resulted from the LA oxidation by overexpressed ROS in cancer cells.

### 3.4. *In vivo* antithrombotic effect of BIL

**Fig. 4:**
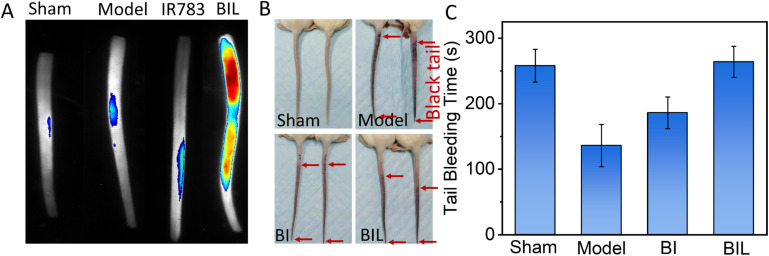
Tail thrombus model of mice induced by carrageen (A and B). (A) Passive targeting of BIL to the tail thrombus after 24 h post BIL injection for the first time without laser irradiation. (B) Antithrombosis effect of BIL on tail thrombus. The red arrows indicate the length of the black colour in the tail. The two tails in the same image were from two representative mice in the same group. (C) Tail bleeding time of tumor-bearing mice after phototherapy treatment for 14 days. The normal mice without tumor were taken as the sham group.

The carrageenan induced tail thrombosis model of tumor-bearing mouse was established to evaluate the antithrombotic effect of BIL (Fig. 4A and B). First, the thrombus targeting of mouse-tail was assessed. The BIL was intravenously injected immediately after intraperitoneal injection of carrageenan. The isolated tails were imaged at 24 h. The tails in the Sham and Model groups showed no fluorescence. The IR783 treated group displayed a weak signal. While the signal intensity of the BIL group was significant. It suggests that the BIL complex has better targeting to the tail thrombus compared to IR783 due to the passive transport of nanomaterials, while IR783 is a small molecule and can be easier cleared out from blood circulation. Then the tail blackening was also observed at 24 h (Fig. 4B and S6†). The whole mice tails showed an obvious black color in the model group, indicating the thrombogenesis. The lengths of black tails in the BI group were a little shorter than that of the carrageenan group. The tail blackening was further obviously inhibited for BIL treatment mice. The results reveal that the NO produced by BIL is crucial for the prevention of thrombogenesis. The carrageenan triggers thrombosis through inflammation induction, platelet activation and endothelial dysfunction. NO has defensive functions towards inflammation, platelet activation and vascular repair through multiple pathways. The synthesized BIL complex can generate a certain amount of NO so that prevents the thrombogenesis.

To evaluate the inhibited hypercoagulation by BIL, we conducted a tail-bleeding assay in which the tumor-bearing mice were first treated with BIL and other formulations followed by irradiation of the tumor seven times and then the bleeding time of the amputated tail was record (Fig. 4C). The tail bleeding time for normal mice was the control. The bleeding time of normal mice was about 256 s. For the tumor-bearing mice in the Model group, the bleeding time was decreased obviously after treatment for 15 days, which was induced by the procoagulant effect of tumor tissues. In contrast, the mice in the BI group and BIL group displayed more prolonged bleeding times of 298 s and 311 s, respectively, suggesting the recovery of the coagulation function of the tumor-bearing mice, which may come from the LA oxidation to NO by overexpression in tumor cells. Significantly, the bleeding time for BIL treated mice was further prolonged to 311 s, almost the same as the mice in the Sham group. It was due to the surge in ROS induced by PDT which oxidized the loaded LA to greater amounts of NO. These results indicated that the released NO could inhibit the activation of platelets, thus suppressing the hypercoagulation and prolonging the bleeding time.

### 3.5. NO-assisted damage to cancer cells *in vitro*

**Fig. 5:**
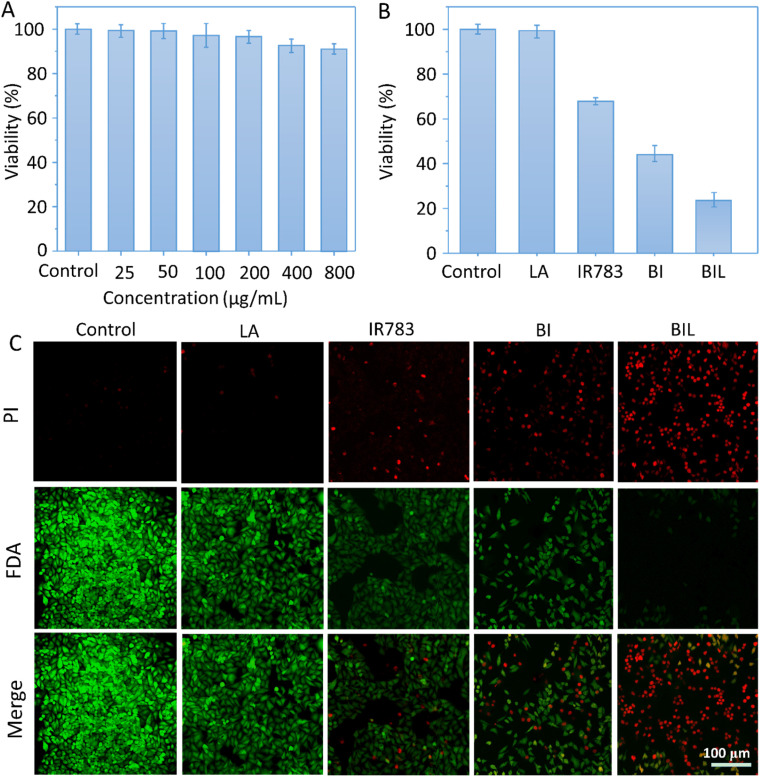
(A) Biocompatibility of BIL. The cells were treated with 0–800 μg mL^−1^ of BIL (at IR783 concentration: 0–144 μg mL^−1^ and LA concentration: 0–96 μg mL^−1^) in the dark. (B and C) Phototherapy induced destruction of HeLa cells in the presence of culture medium (control), LA, IR783, BI and BIL (600 μg mL^−1^, at IR783 concentration: 108 μg mL^−1^ and LA concentration: 72 μg mL^−1^) with laser irradiation for 5 min evaluated by CCK-8 assay and CLSM (viable cells were stained green with FDA, and dead cells were stained red with PI). Scale bar: 100 μm.

To evaluate the therapeutic outcomes, we first investigated the *in vitro* biocompatibility and anticancer capacity of BIL and other counterparts (Fig. 5A–C). It was biocompatible over 0–800 μg mL^−1^ with the cell viability above 90% for BIL treated cells, providing a biocompatible concentration range for the following cellular and *in vivo* experiments. The free LA showed no significant cytotoxicity to cells while the viability of free IR783 and BI treated cells dropped to 68% and 44% under 808 nm laser irradiation. Whereas, the cells in the BIL treatment group were ablated to 22% with laser. These results demonstrated that the introduction of LA further raised the killing effects of the BIL particles. To display the killing capacity visually, live–dead cell staining was performed through co-staining the cells with FDA (green fluorescence) and PI (red fluorescence) which was observed with CLSM. The cells showed no obvious death after treatment with free LA under irradiation while they showed slight cell death after treatment with IR783 under irradiation for 5 min compared to the control group. However, the cells in the BIL treatment groups under irradiation showed apparent death, suggesting the increased therapeutic efficacy of the particles, which probably resulted from synergy between LA and IR783. The generated NO from oxidation of LA triggers the cancer cell apoptosis pathway and induces a severe cell death.

### 3.6. NO-assisted anticancer performance *in vivo*

**Fig. 6:**
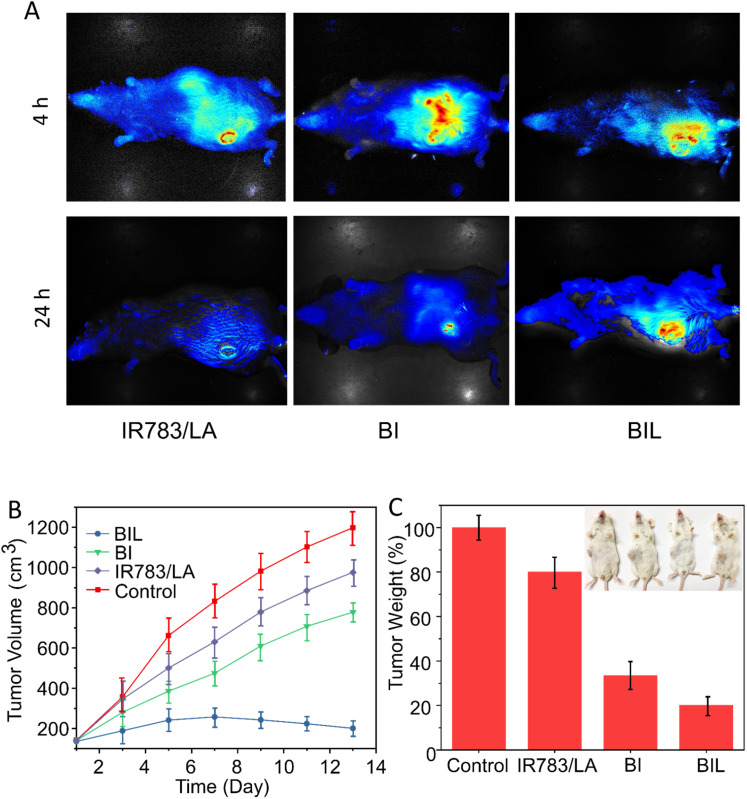
(A) *In vivo* fluorescence of mice bearing implanted U14 cancer after the intravenous injection of free IR783/LA, BI or BIL at 4 and 24 h with 808 nm laser irradiation. (B) The relative tumor volume curves and (C) weights of excised tumors.

We next evaluated the effect on tumor accumulation of the LA-assisted phototherapy with BIL using the U14 tumor-bearing mouse model. At 12 h post injection of free IR783, BI and BIL, the tumor tissues in each group were illuminated by the 808 nm NIR laser for 5 min and the fluorescence images of IR783 were obtained at 4 and 24 h. As shown in Fig. 6A, the fluorescence in the free IR783 group reached a maximum value at 4 h and then rapidly attenuated within 24 h, indicating a more rapid metabolism and elimination from the mice compared with the BI and BIL groups. In addition, the BIL group showed a more intense signal than the BI group at 24 h due to the induction of LA which leads to a longer accumulation in the tumor for the particles.

*In vivo* experiments were performed to confirm the cancer therapeutic effect of BIL based on the antiplatelet-assisted strategy. As shown in Fig. 6B and C, the tumor growth in mice after injection with free IR783/LA, BI or BIL was inhibited to different extents as compared to that of the control mice. Tumors in the free IR783/LA injected groups were fast-growing, which might be due to the rapid clearance and less favorable tumor accumulation of small molecules. Moreover, the BI injection induced a moderate inhibition of tumor growth. Note that BIL exhibited the best phototherapy efficiency as it almost completely ablated the tumors (Fig. S7†). Similarly, the tumor weight in the BIL treatment group is much lighter than that of the other groups, indicating the high efficacy of the NO-assisted phototherapy.

## 4. Conclusions

In conclusion, novel NO release proteinic nanoparticles for antiplatelet-assisted cancer phototherapy were successfully developed to effectively destroy cancer cells. Through the oxidation of guanidine on the side-chain of LA by ROS produced from activated IR783 molecules under NIR laser irradiation, NO released locally in the tumor inhibited platelet activation, resulting in enhanced perfusion of nanoparticles to tumor tissues, reduction of hypercoagulability and potent therapeutic efficacy without systemic bleeding risk. In addition, the released NO was readily oxidized to peroxynitrite that was highly reactive and hypertoxic to cancer cells, increasing the cell killing capacity of the nanoparticles. The present work not only provides important hints to develop a NO-assisted phototherapy but also offers positive insight to expand this strategy to other therapeutic approaches, such as chemical therapy and radiotherapy through enhancing the perfusion of drugs or radiosensitizers.

## Author contributions

Rui Liu: methodology; investigation. Baofeng Xu: methodology; investigation. Zhifang Ma: conceptualization, data curation, funding acquisition, writing – original draft, visualization. Hongbo Ye: methodology; investigation. Xinghua Guan: methodology, investigation. Yue Ke: data curation, methodology. Zehong Xiang: data curation, methodology. Qiang Shi: resources, writing – review & editing, supervision, project administration, funding acquisition.

## Conflicts of interest

The authors declare no competing financial interests.

## Supplementary Material

RA-012-D2RA05438H-s001
