# Supplementary material for: Controlled release of nitric oxide for enhanced tumor drug delivery and reduction of thrombosis risk
Source: RSC Adv. 2022 Nov 11;12(50):32355–64. doi: 10.1039/d2ra05438h (PMC9650582; doi:10.1039/d2ra05438h)
Supplement: RA-012-D2RA05438H-s001 [file RA-012-D2RA05438H-s001.pdf]

Electronic Supplementary Material (ESI) for RSC Advances.
This journal is © The Royal Society of Chemistry 2022

Controlled Release of Nitric Oxide for Enhanced Tumor Drug Delivery and Reduction of Thrombosis Risk

Rui Liu, Baofeng Xu, Zhifang Ma, Hongbo Ye, Xinghua Guan, Yue Ke, Zehong Xiang and Qiang Shi

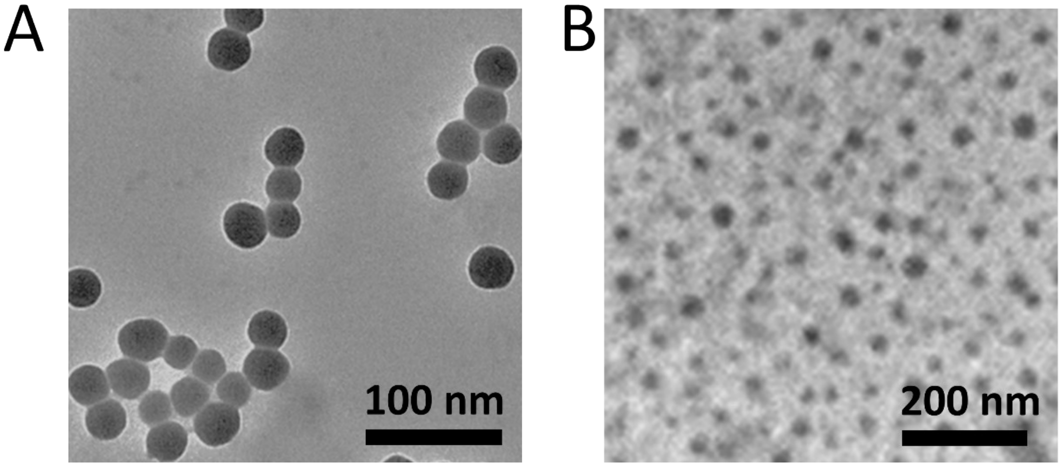

Figure S1. The TEM images of BI and BL.

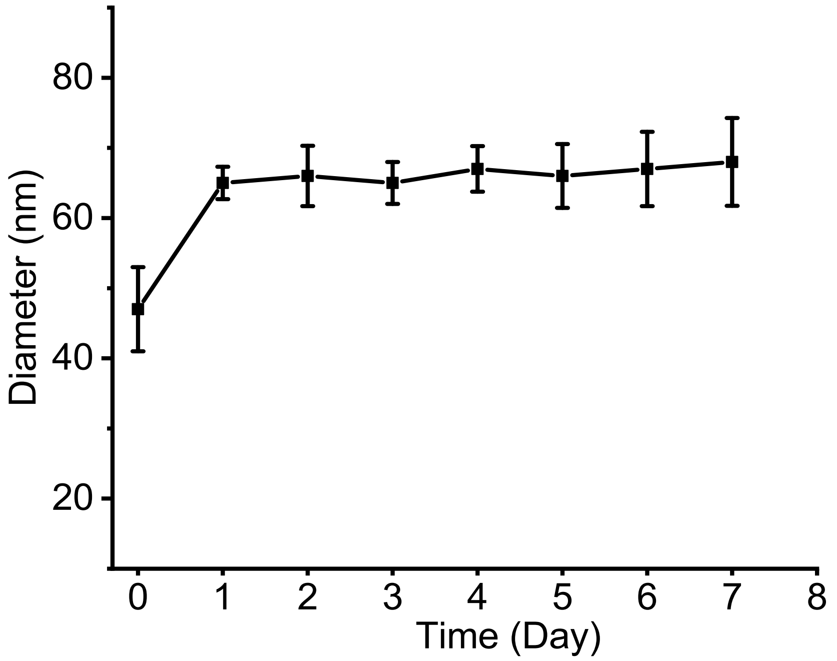

Figure S2. DLS profile of BIL for samples, 24 h and 7 days. The data for day 0 and day 1 were measured after fresh synthesis and 24 h, respectively.

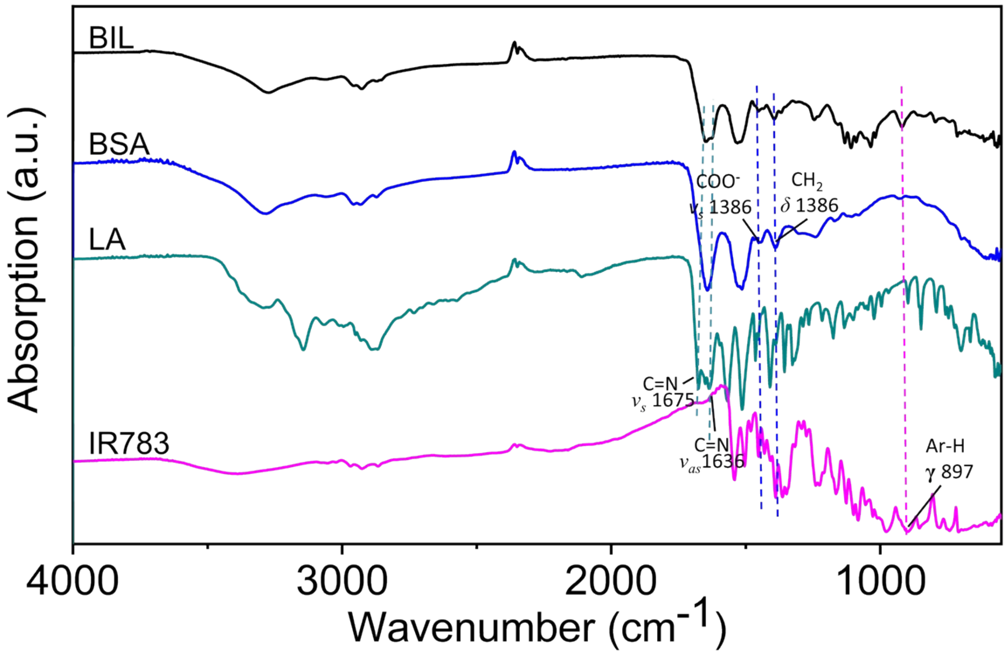

Figure S3. FTIR spectra of IR783, LA, BSA and BIL. The peak assignments in FTIR spectra were marked by coloured dotted line.

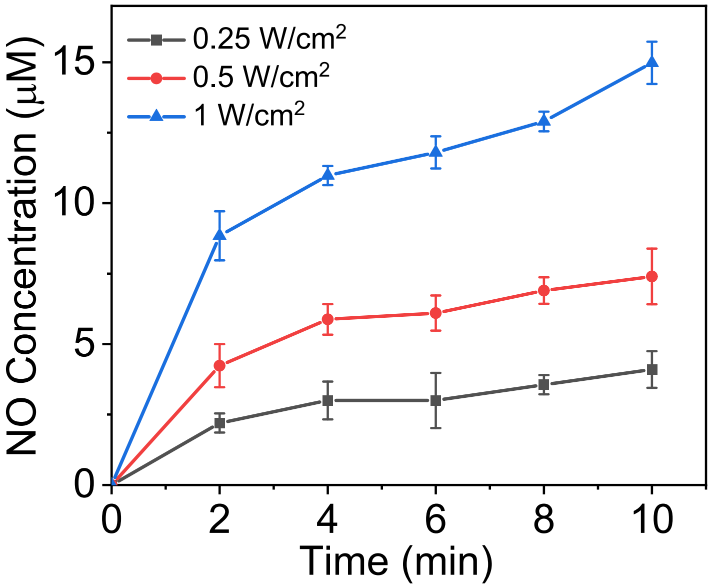

Figure S4. The NO generation under difference laser power density (0.25 W/cm^2, 0.5 W/cm^2 and 1 W/cm^2) within 10 min.

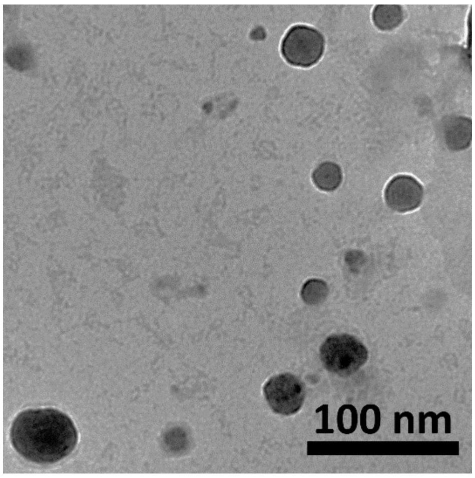

Figure S5. TEM image of BIL after irradiated by 808 nm laser for 10 min (1.0 W/cm^2).

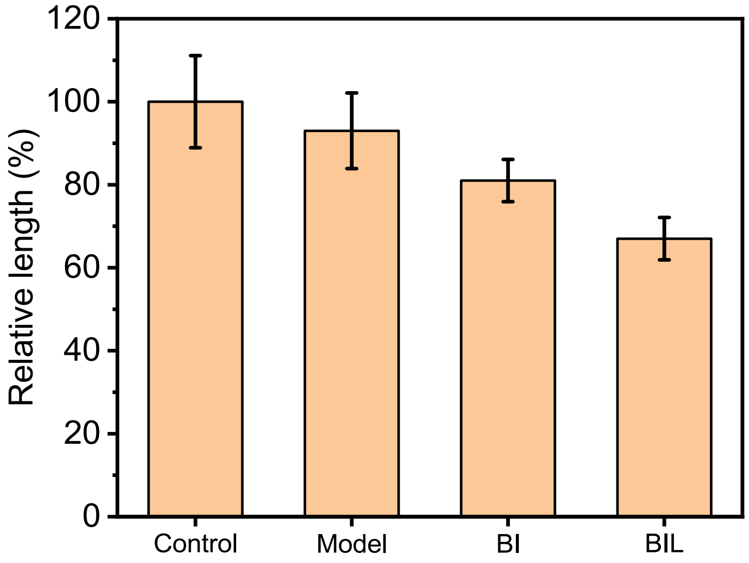

Figure S6. The relative length of the black tail of tumor-bearing mice after establishment of the tail thrombus model.

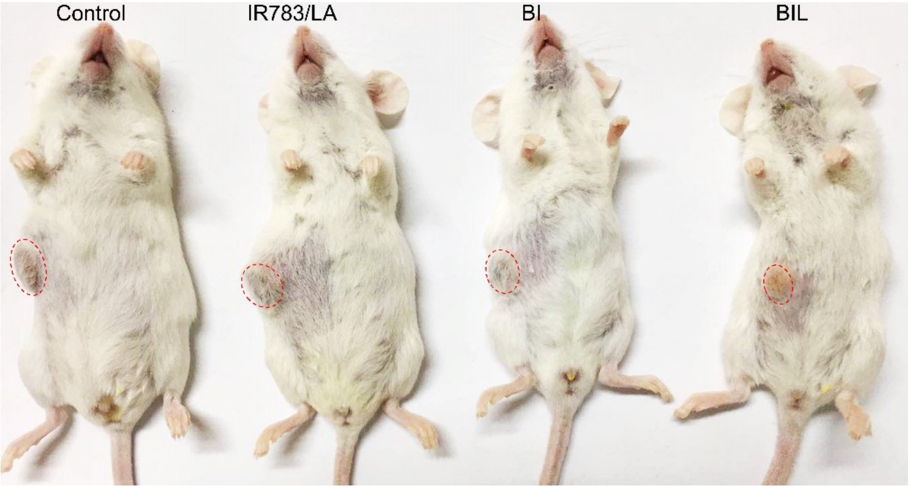

Figure S7. The representative image of the tumor-bearing mice. The circle indicates the tumor outline.
